# Exposure of the cytoplasm to low-dose X-rays modifies ataxia telangiectasia mutated-mediated DNA damage responses

**DOI:** 10.1038/s41598-021-92213-z

**Published:** 2021-07-05

**Authors:** Munetoshi Maeda, Masanori Tomita, Mika Maeda, Hideki Matsumoto, Noriko Usami, Kyo Kume, Katsumi Kobayashi

**Affiliations:** 1 Proton Medical Research Division, Research and Development Department, The Wakasa Wan Energy Research Center, WERC, 64-52-1 Nagatani, Tsuruga, Fukui 914-0192 Japan; 2 Radiation Safety Research Center, Nuclear Technology Research Laboratory, Central Research Institute of Electric Power Industry, CRIEPI, 2-11-1 Iwado Kita, Komae, Tokyo 201-8511 Japan; 3 Department of Experimental Radiology and Health Physics, Faculty of Medical Sciences, University of Fukui, 23-3 Matsuoka-Shimoaitsuki, Eiheiji-cho, Fukui, 910-1193 Japan; 4 Photon Factory, Institute of Materials Structure Science, High Energy Accelerator Research Organization, KEK, 1-1 Oho, Tsukuba, Ibaraki 305-0801 Japan

**Keywords:** Cell death, Cell signalling, DNA damage and repair

## Abstract

We recently showed that when a low X-ray dose is used, cell death is enhanced in nucleus-irradiated compared with whole-cell-irradiated cells; however, the role of the cytoplasm remains unclear. Here, we show changes in the DNA damage responses with or without X-ray microbeam irradiation of the cytoplasm. Phosphorylated histone H2AX foci, a surrogate marker for DNA double-strand breaks, in V79 and WI-38 cells are not observed in nucleus irradiations at ≤ 2 Gy, whereas they are observed in whole-cell irradiations. Addition of an ataxia telangiectasia mutated (ATM) kinase inhibitor to whole-cell irradiations suppresses foci formation at ≤ 2 Gy. *ABL1* and *p73* expression is upregulated following nucleus irradiation, suggesting the induction of p73-dependent cell death. Furthermore, *CDKN1A* (*p21*) is upregulated following whole-cell irradiation, indicating the induction of cell cycle arrest. These data reveal that cytoplasmic radioresponses modify ATM-mediated DNA damage responses and determine the fate of cells irradiated at low doses.

## Introduction

Since the discovery of X-rays by Prof. Wilhelm Conrad Röntgen in 1895, many scientists have studied the nature of radiation. Ionizing radiation (IR) exerts significant biological effects via imperceptible amounts of energy, compared with the energy deposition of life forms by other sources, such as thermal energy. For example, even if a man is exposed to a 50% lethal dose of X-ray (4 Gy, or 4 J kg^−1^), the rise in body temperature is only ~ 0.001 °C. IR deposits energy randomly, thereby causing damage to all molecules in the cell. Ionized molecules are highly reactive, disrupting the structure of macromolecules. However, as most biomolecules, for example water, mRNA, and proteins, have multiple copies and a continuous, rapid turnover, they are not considered to contribute significantly to radiation effects. In contrast, DNA, which is central to all cellular functions, has only two copies in a cell and a very limited turnover. Therefore, the consequences associated with permanent DNA damage are serious and often lethal for the cell. In the 1970s, DNA has been experimentally proven as a major target for radiation-induced cell death. In the case of irradiating individual cells with small polonium needles producing short-range α-particles, cell death is not observed with high-dose irradiation of the plasma membrane and cytoplasm^1^. In contrast, cell death is induced when the nucleus is irradiated with one or two α-particles^1^. In other experiments using radioactively-labeled compounds, cell death closely correlates with the dose administered to the nucleus, but not with doses administered to either the plasma membrane or the cytoplasm^2,3^. Thus, it is generally accepted that majority of biologically important damage produced by IR occurs to nuclear DNA, either by direct ionization or through reactions with free radicals produced nearby. Therefore, the biological consequences of radiation exposure, including cell death, are highly influenced by pathways within the DNA damage response (DDR) system. Ataxia telangiectasia mutated (ATM) serine/threonine kinase is a key regulator of the DDR system^4,5^. Nuclear ATM responds to DNA double-strand breaks (DSBs), and upregulates cell cycle checkpoint pathways, including cell cycle arrest and DNA repair^4,5^. Although ATM has long been considered a nuclear protein that functions in response to genotoxic stress, recent studies have revealed that IR exposure induces cytoplasmic ATM monomerization, and these monomers translocate to the nucleus^6–10^. However, the role of the cytoplasm in the cellular response to IR remains unclear, including in the nucleo-shuttling of ATM from the cytoplasm during a DDR. To elucidate the precise cellular responses initiated by the deposition of IR energy at the local site of cells, such as the nucleus or cytoplasm, we developed a synchrotron radiation (SR)-based X-ray microbeam irradiation system^11,12^. Owing to the very low divergence of SR X-rays, our system provides custom-sized X-ray microbeams larger than 5 × 5 µm using a precise four-bladed slit system. X-ray energy is 5.35 keV, which is high enough to penetrate cell samples uniformly. Using X-ray microbeams, we compared the survival of whole-cell- and nucleus-irradiated Chinese hamster V79 cells. Higher cell death was observed in the latter, compared with the former, even though the amount of DNA damage in the nucleus was the same irrespective of cytoplasmic irradiation^13^. Moreover, we demonstrated that the death of bystander V79 cells, defined as nonirradiated V79 cells located near irradiated V79 cells, was clearly higher following nuclear irradiation with low-dose X-ray microbeams than after whole-cell irradiation^14,15^. In addition, we confirmed a similar response in the normal human fibroblast WI-38 cell line^16,17^. Our findings indicate that the DDR is affected by the site of radiation energy deposition within cells. Cell death induced through irradiation of the nucleus is rescued via cytoplasmic radiation energy deposition. As described above, the biological consequences of radiation exposure, including cell death, are known to be influenced by components of the DDR. Energy deposition in the cytoplasm may play an important role in the DDR. However, most mechanisms underlying the cellular response to cytoplasmic irradiation remain unclear.

Here, we discuss the intracellular nuclear- and cytoplasmic-ATM responses during a DDR, elicited by local energy deposition in the cell using our SR X-ray microbeam irradiation technique. We demonstrated that in cells exposed to low doses of X-rays, energy deposition in the cytoplasm was required for induction of phosphorylation of H2AX, which occurs during the early stages of the DDR. Moreover, this cytoplasmic response was inhibited by the addition of a specific ATM inhibitor. Furthermore, gene expression analysis revealed that a cell death pathway was induced when the cytoplasm was not irradiated, whereas DNA repair cascades were promoted when the cytoplasm was irradiated. The data indicate that low-dose X-ray irradiation of the cytoplasm modifies the ATM-mediated DDR, and hence, determines the fate of the cells.

## Results

### Induction of cell death by cytoplasm irradiation

**Figure 1:**
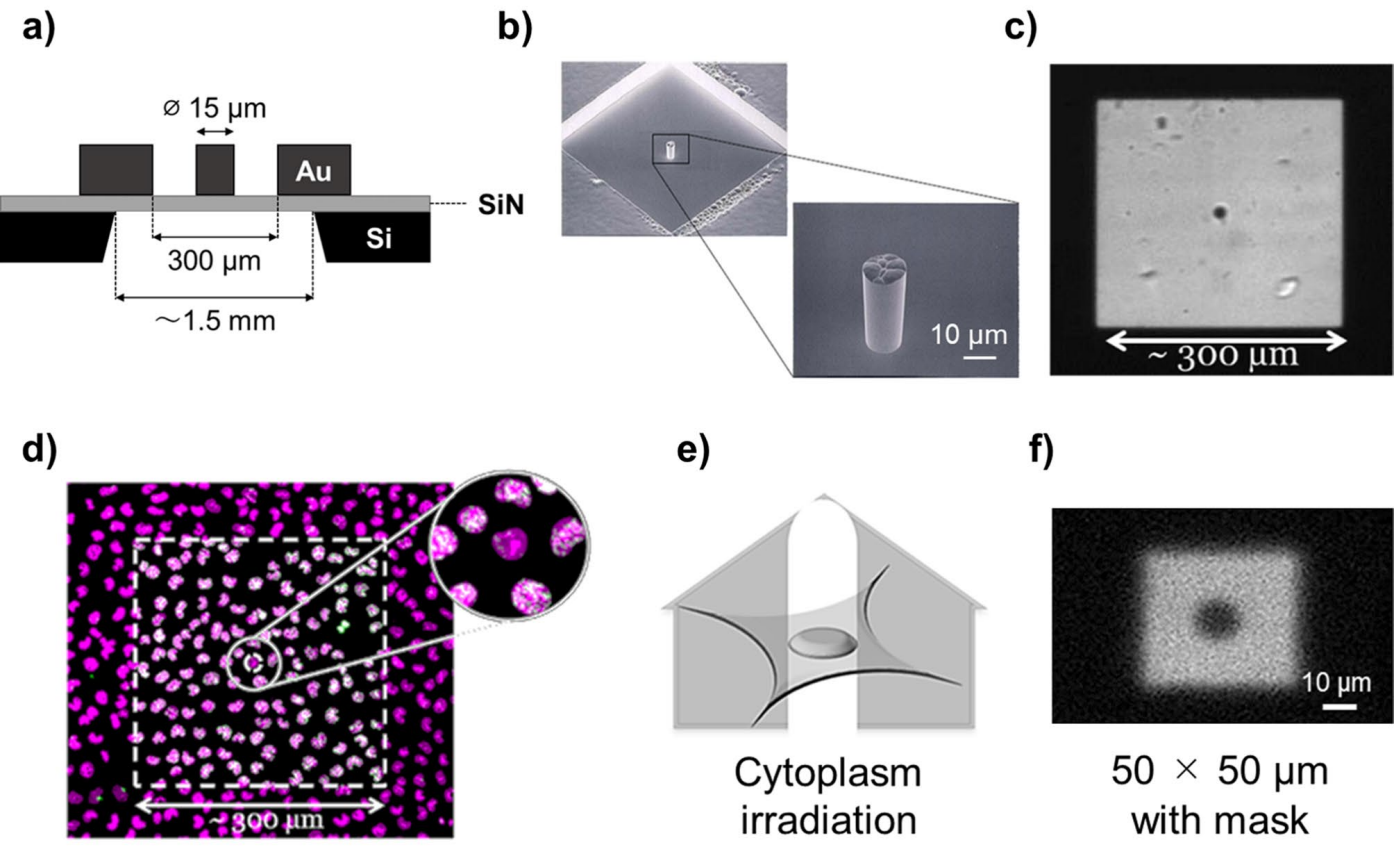
A method for cytoplasm irradiation using synchrotron radiation X-ray microbeams. (**a**) Cross-sectional illustration of a custom-designed X-ray mask. (**b**) Scanned electron microscopy analysis showing the gold post at the center of the X-ray mask responsible for shielding the nucleus from X-ray microbeams. (**c**) Shape of the X-ray beam formed in combination with the X-ray mask and a 500 × 500 µm beam, as visualized using a scintillator. (**d**) Accumulation of phosphorylated histone H2AX (γ-H2AX) foci in V79 cells irradiated with 5 Gy X-ray beam shown in (**c**). Propidium iodide staining and γ-H2AX foci in the captured image are presented as magenta and green signals, respectively. (**e**) Representation of cytoplasm irradiation in a single targeted cell. (**f**) Shape of a 50 × 50 µm beam penetrating the mask used to irradiate the cytoplasm of V79 cells.

**Figure 2:**
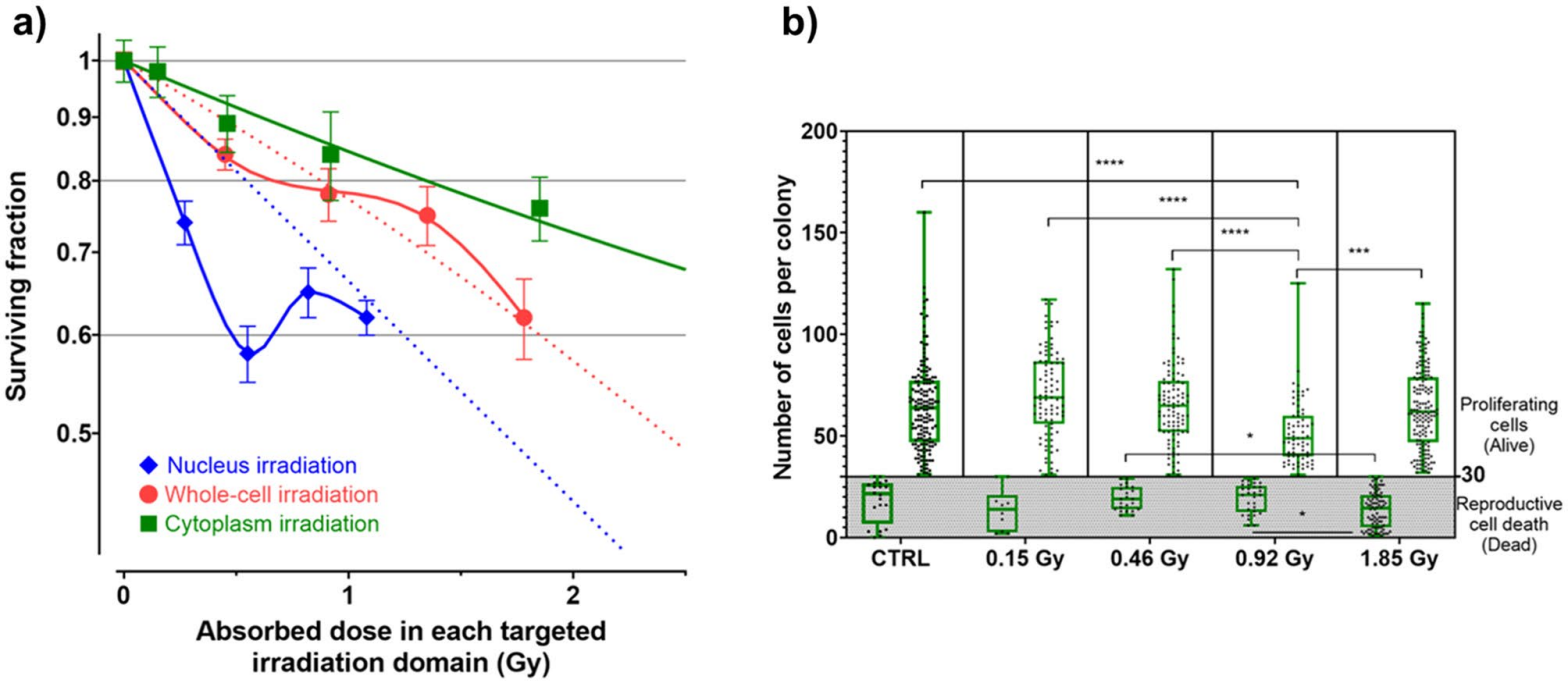
Surviving fraction of V79 cells irradiated with differently sized synchrotron radiation X-ray microbeams. (**a**) Surviving fractions of V79 cells irradiated with a 50 × 50 µm X-ray beam with the X-ray mask (filled squares; cytoplasm irradiation) were plotted as a function of the absorbed dose in the cytoplasm. Standard errors were calculated using Eq. (4). The total number of colonies counted (N in Eq. 4) was 181, 100, 125, 100, 245 at 0 Gy (non-irradiated control), 0.15 Gy, 0.46 Gy, 0.92 Gy, and 1.9 Gy irradiation, respectively. Surviving fractions of V79 cells irradiated with 10 × 10 µm (filled diamonds; nucleus irradiation) and 50 × 50 µm (filled circles; whole-cell irradiation) X-ray beams were obtained from our previous work^13^. Each data point represents the mean of at least three independent experiments. Dotted lines show the linear-quadratic fitting based on data for higher radiation doses. p-values of cytoplasmic irradiation are shown in Supplementary Table S1 online, and the R^2^ of survival curve of cytoplasmic irradiation was 0.9930. (**b**) The distributions of the number of cells per colony in the nonirradiated control (CTRL) and cytoplasm-irradiated cell groups are shown by box-and-whisker plots with individual data points. Colonies with more than 30 cells per colony after 60 h of irradiation were considered viable. The boxes indicate the 25th–75th percentile, the line in the boxes indicates the median, and the whiskers indicate the range (minimum to maximum) of the data. p-values (*ns* not significant, *p < 0.05, **p < 0.01, ***p < 0.001, ****p < 0.0001) calculated with Tukey's multiple comparison test. p-values for each comparison pair are also shown in Supplementary Table S2 online.

To assess the cellular response induced by X-ray irradiation of the cytoplasm alone, the nucleus was selectively shielded using a gold mask (Fig. 1a,b). To verify the shielding ability of the mask, V79 cells were targeted with a 300 × 300 µm beam with a 15-μm diameter gold post located at its center (Fig. 1c). Using this method, the nucleus of targeted cells was not irradiated, while its cytoplasm and other cells in the irradiated field were exposed to 5 Gy X-rays. As shown in Fig. 1d, phosphorylated histone H2AX (γ-H2AX) foci were absent from the nucleus of shielded cells, but abundant in the remaining cells. From these results, we confirmed that the X-ray mask successfully shielded target cell nuclei. Next, we measured the surviving fraction of the cytoplasm-irradiated V79 cells using a 50 × 50 µm beam with the X-ray mask shielding the beam centrally (Fig. 1e,f). Figure 2a shows the dose-survival relationship obtained following cytoplasmic irradiation, as well as the data from nucleus- and whole-cell-irradiated cells from our previous study^13^. The distribution of the number of cells per colony for each data point of cytoplasmic irradiation is shown in Fig. 2b. From the Tukey's multiple comparison test, the number of cells per colony of viable cells (defined as living, proliferating cells) in the 0.92 Gy irradiated group was found to be significantly smaller than that in the control group and all other irradiated groups (p < 0.0001 for all instances except against 1.85 Gy, where p < 0.001). Furthermore, the number of cells per colony of dead cells (defined by reproductive death of the cells) in the 0.46 and 0.92 Gy irradiated groups was significantly larger (p < 0.05) than that in the 1.85 Gy irradiated group. The changes in cell proliferation due to cytoplasmic irradiation were most markedly induced at 0.92 Gy (Fig. 2b). However, the surviving fraction of cytoplasm-irradiated V79 cells decreased exponentially (R^2^ = 0.993) with an increase in the doses absorbed by the cytoplasm. Thus, the changes in cell proliferation due to cytoplasmic irradiation would have little effect on the determination of cell fate.

### Association between the cell irradiation site and γ-H2AX accumulation

**Figure 3:**
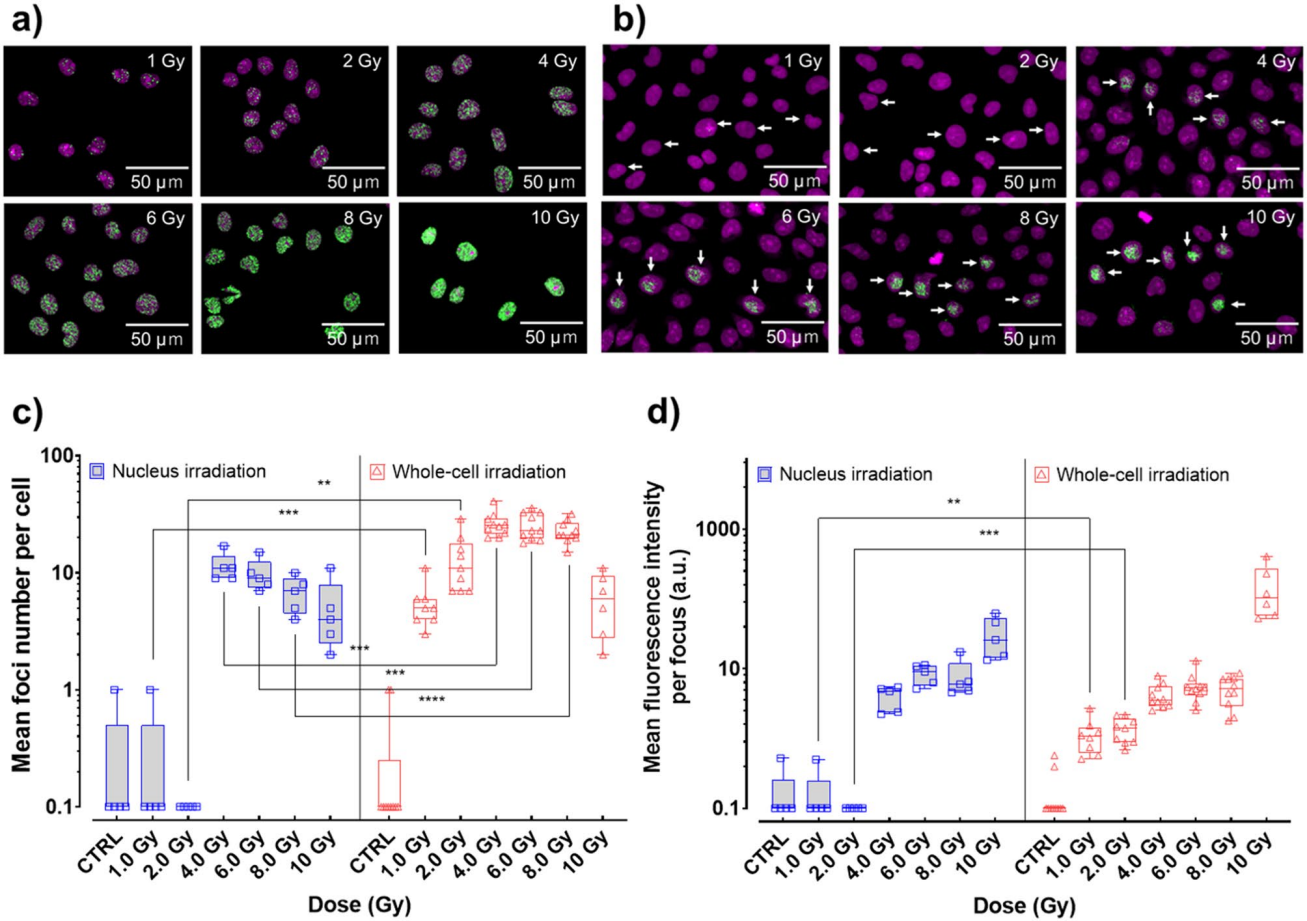
Accumulation of phosphorylated histone H2AX (γ-H2AX) foci in V79 cells after 30 min of nucleus or whole-cell irradiation. γ-H2AX foci formation following the irradiation of V79 whole cells (**a**) or V79 nuclei (**b**). White arrows in (**b**) identify irradiated cells. Propidium iodide staining and γ-H2AX foci in the captured images are presented as magenta and green signals, respectively. During imaging analysis, the mean foci number in irradiated V79 cells (**c**) and the mean fluorescence intensity at a focus in V79 cells (**d**) was measured using Image-Pro Plus 7.0. CTRL, nonirradiated control cells. To make a quantitative comparison, analysis was performed using images acquired under the same condition in the same batch of experiments. Results are shown by box-and-whisker plots with individual data points. The boxes indicate the 25–75th percentile, the line in the boxes indicates the median, and the whiskers indicate the range (minimum to maximum) of the data. p-values (*ns* not significant, *p < 0.05, **p < 0.01, ***p < 0.001, ****p < 0.0001) in (**c**) and (**d**) calculated by Student's *t* test. p-values for each comparison pair are also shown in Supplementary Tables S3 and S4 online.

**Figure 4:**
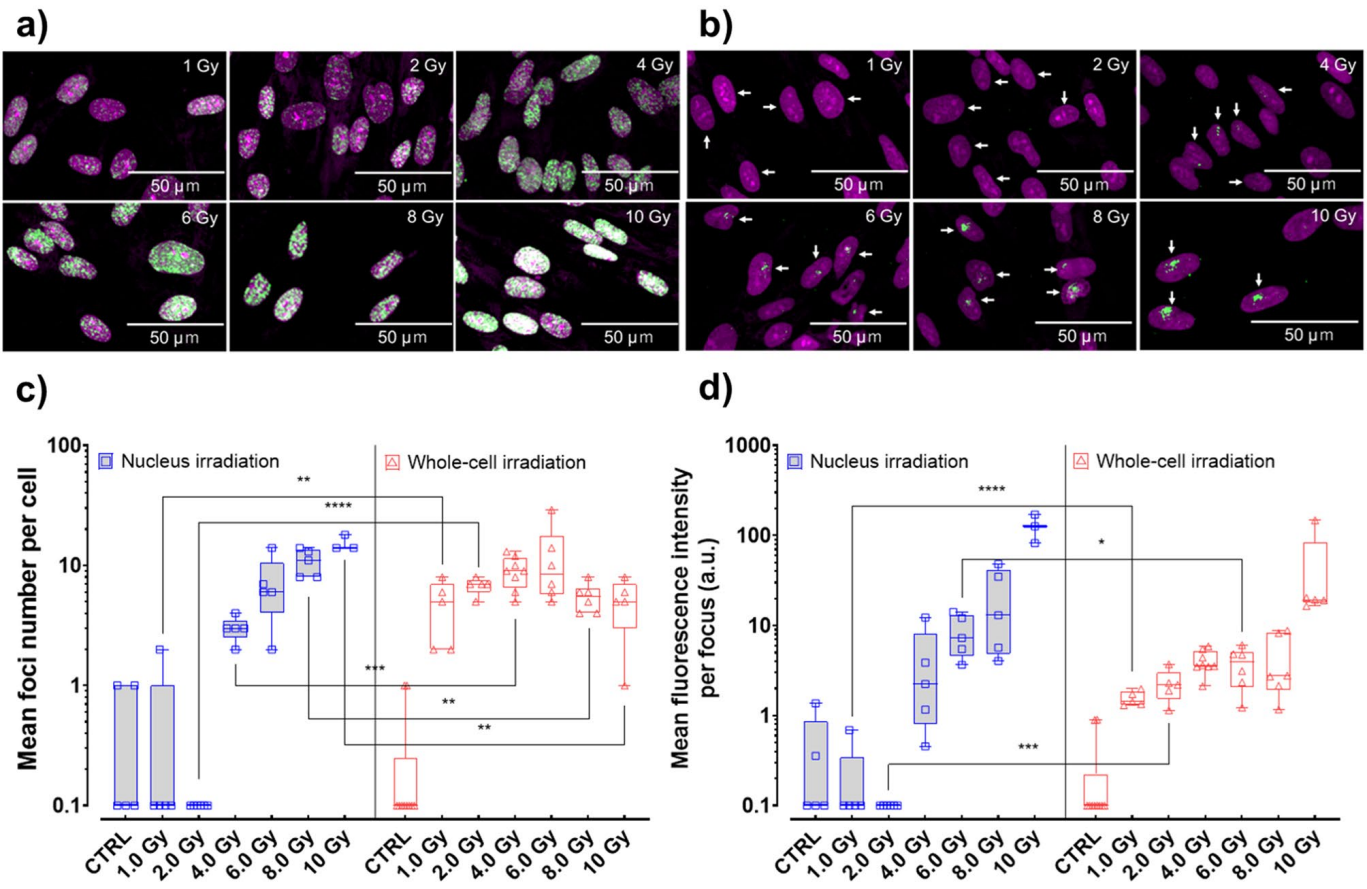
Accumulation of phosphorylated histone H2AX (γ-H2AX) foci in WI-38 cells after 30 min of nucleus or whole-cell irradiation. γ-H2AX foci formation following the irradiation of WI-38 whole cells (**a**) or WI-38 nuclei (**b**). White arrows in (**b**) identify irradiated cells. Propidium iodide staining and γ-H2AX foci in the captured images are presented as magenta and green signals, respectively. During imaging analysis, the mean foci number in irradiated WI-38 cells (**c**) and the mean fluorescence intensity at a focus in WI-38 cells (**d**) are measured using Image-Pro Plus 7.0. CTRL, nonirradiated control cells. To make a quantitative comparison, analysis was performed using images acquired under the same condition in the same batch of experiments. Results are shown by box-and-whisker plots with individual data points. The boxes indicate the 25–75th percentile, the line in the boxes indicates the median, and the whiskers indicate the range (minimum to maximum) of the data. p-values (*ns* not significant, *p < 0.05, **p < 0.01, ***p < 0.001, ****p < 0.0001) in (**c**) and (**d**) calculated with Student's *t* test. p-values for each comparison pair are also shown in Supplementary Tables S5 and S6 online.

We assessed the dose-dependent accumulation of γ-H2AX foci in V79 cells irradiated with 130 × 130 µm beams at doses of 1–10 Gy. These foci served as a record of the induction of DNA DSB repair cascades. As shown in Fig. 3a, γ-H2AX foci were observed following whole-cell irradiation at doses ≥ 1 Gy. In contrast, Fig. 3b shows a dose-dependent increase in γ-H2AX fluorescence in cells whose nuclei were selectively targeted with a 10 × 10 µm beam. Although the same dose was administered to the nuclei in both the cases, γ-H2AX foci were not observed in cells whose nuclei were irradiated at doses ≤ 2 Gy. Next, we counted the number of foci in irradiated cells (Fig. 3c), also measuring the fluorescence intensity of each focus (Fig. 3d). The level of induction of foci in nuclei irradiated with 1–2 Gy X-ray microbeams showed no significant difference compared with that of non-irradiated cells, whereas the induction level increased with dose escalation in whole-cell irradiation at doses between 1 and 4 Gy. Although in nucleus and whole-cell irradiation, the number of foci per cell appeared saturated with dose escalation and then decreased at 10 Gy, fluorescence intensities of the foci dose-dependently increased in both cases. In particular, the increase in fluorescence intensity at 10 Gy is remarkable. These results indicate that multiple foci in close proximity had fused with each other, resulting in a decrease in the number of apparent foci at high doses. In addition, the number of foci per cell was lower in the nucleus irradiation than in the whole-cell irradiation at 4–8 Gy (p < 0.001 for 4 and 6 Gy, p < 0.0001 for 8 Gy). The number of photons incident to the nucleus was almost same for both whole-cell and nucleus irradiation at the same dose. However, since nucleus irradiation was carried out using a beam smaller than the nucleus, the density of the resulting DNA damage might be slightly higher than in case of whole-cell irradiation. These might be the reasons why large and little bright foci were observed in the nucleus irradiation. To verify whether this phenomenon could be observed in other mammalian cells, we irradiated the normal human fibroblast WI-38 cell line with 200 × 200 µm beams at doses between 1 and 10 Gy. As shown in Fig. 4a, γ-H2AX foci were found at doses ≥ 1 Gy following whole-cell irradiation. Nucleus-specific irradiation confirmed the results observed in V79 cell line, as foci could not be observed in cells irradiated at doses ≤ 2 Gy (Fig. 4b). As shown in Fig. 4c,d, a quantitative analysis of WI-38 cells showed almost the same trend as that obtained using V79 cells. Since a 5 × 5 μm beam, a quarter of that used for irradiation of the nucleus of V79 cells, was used for WI-38 nucleus irradiation, the foci were concentrated in a small area and the spatial resolution of the fluorescent signal became lower than that noted using V79 cells. Thus, the dose-dependent saturation and reduction in the number of foci per cell in nucleus-irradiated WI-38 cells could not be observed in V79 cells.

**Figure 5:**
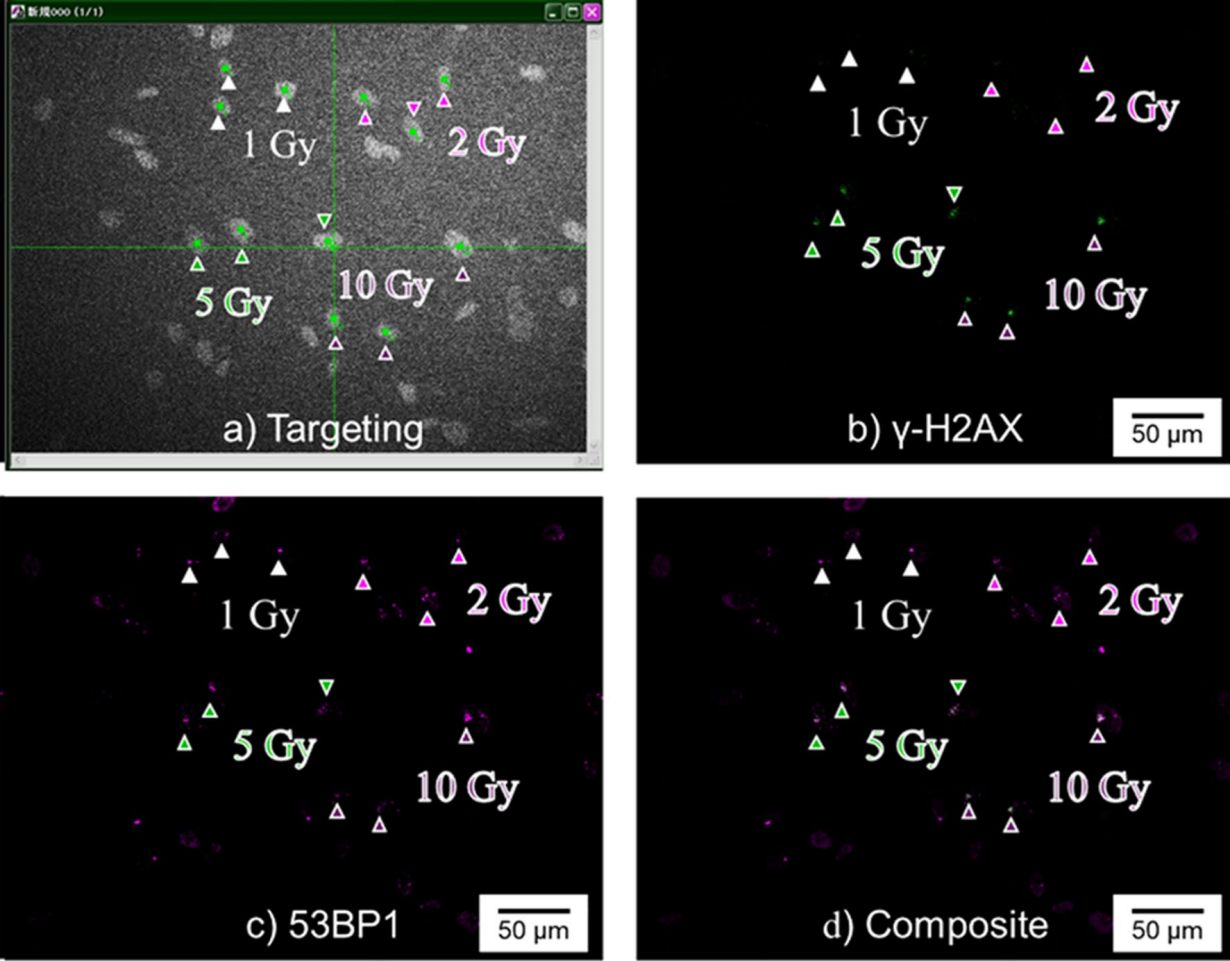
Accumulation of p53-binding protein 1 (53BP1) and phosphorylated histone H2AX (γ-H2AX) foci in nucleus-irradiated WI-38 cells after 30 min of irradiation. (**a**) The nucleus of each of the three WI-38 cells on the same dish was irradiated with 1, 2, 5, or 10 Gy 5 × 5 µm beams. Green squares symbolize the targeted positions. (**b**) γ-H2AX foci formation at the irradiated sites. (**c**) 53BP1 foci formation at the irradiated sites. (**d**) Composite of (**b**) and (**c**). The arrows in each panel indicate the position of irradiated nuclei. 53BP1 and γ-H2AX foci formations in the captured images are presented as magenta and green signals, respectively. The dose deposited in each nucleus is shown in each panel.

**Figure 6:**
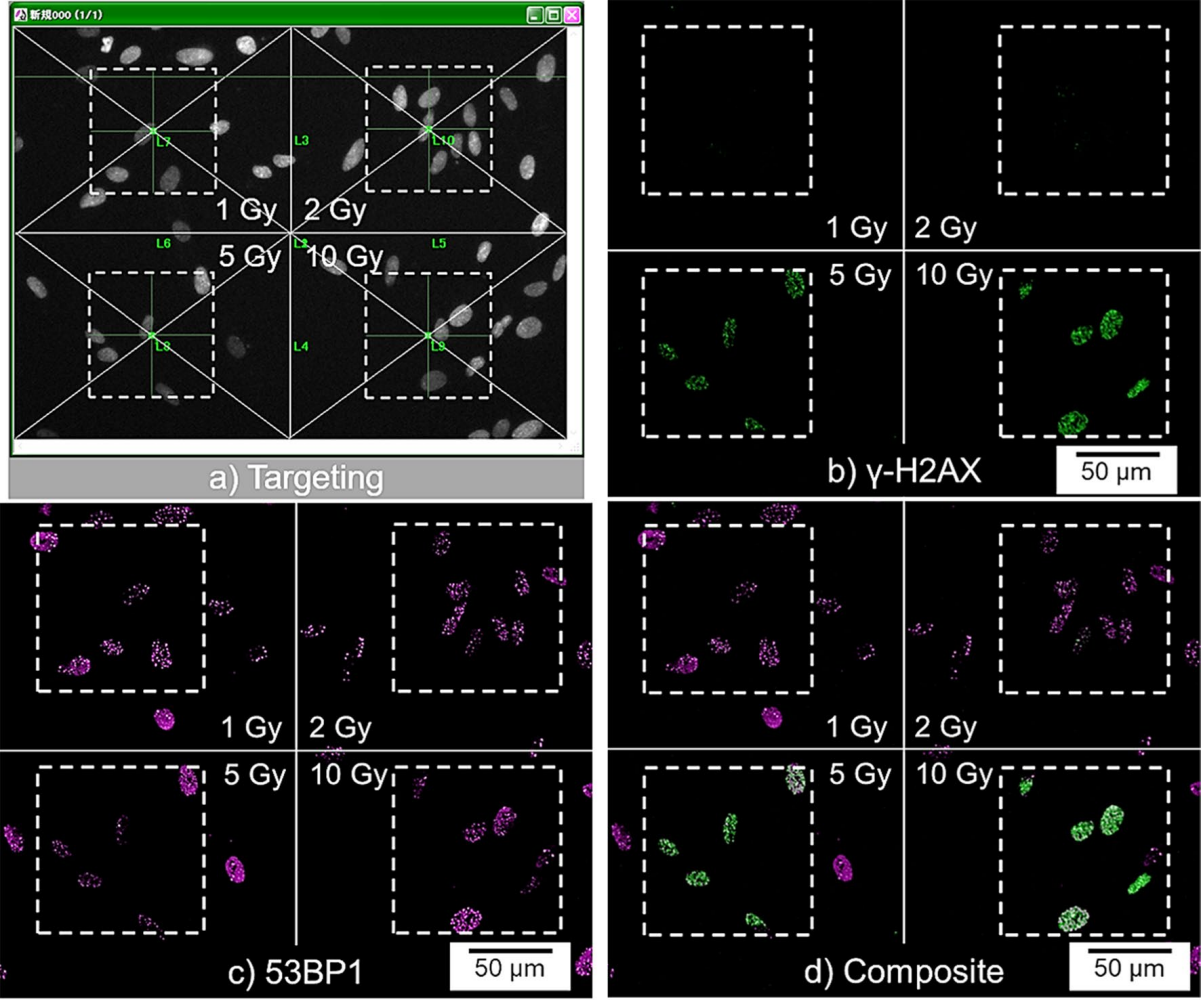
Accumulation of p53-binding protein 1 (53BP1) and phosphorylated histone H2AX (γ-H2AX) foci in whole-cell-irradiated WI-38 cells in the presence of an ATM inhibitor following 30 min irradiation. (**a**) An acquired image frame was divided into four, with WI-38 cells existing at the center of each area irradiated with 100 × 100 µm beams of 1, 2, 5, or 10-Gy. (**b**) γ-H2AX foci formation at the irradiated sites. (**c**) 53BP1 foci formation at the irradiated sites. (**d**) Composite of (**b**) and (**c**). 53BP1 and γ-H2AX foci formations in the captured images are presented as magenta and green signals, respectively. White dotted squares represent the 100 × 100 µm beam. The X-ray doses deposited in each area are shown in each panel.

To eliminate the possibility that the lack of γ-H2AX foci formation observed for the low-dose nucleus irradiation was due to the failure of microbeam targeting, we examined the accumulation of p53-binding protein 1 (53BP1) in cells with nucleus irradiation between 1 and 10 Gy doses (Fig. 5a). As shown in Fig. 5b–d, 53BP1 foci formation was observed at all doses, while γ-H2AX foci formation was not induced at doses ≤ 2 Gy. Irradiating the nucleus with 1–10 Gy microbeams was reliably demonstrated to produce DSBs, as 53BP1 accumulates at the DSB site in an ATM serine/threonine kinase-independent manner, unlike H2AX phosphorylation^18^. These findings clearly indicate that energy deposition in the nucleus, together with the cytoplasm, is necessary to activate the DDR, especially the DSB repair process, at low radiation doses. Furthermore, energy deposition in the cytoplasm was indicated as necessary for activating ATM functions, such as H2AX phosphorylation in the region targeted with low-dose radiation. To verify whether ATM controls H2AX phosphorylation in the low-dose region, WI-38 cells were irradiated with 100 × 100 µm microbeams in the presence of an ATM-specific inhibitor (20 μM) (Fig. 6a). As shown in Fig. 6b–d, DNA damage was induced at all doses, as indicated by the observation of 53BP1 foci, while γ-H2AX foci formation was almost completely suppressed by the addition of an ATM inhibitor at doses ≤ 2 Gy. In contrast, as shown in Figs. 3a and 4a, significant γ-H2AX foci formation was observed following whole-cell irradiation with 1–10 Gy X-ray microbeams in the absence of an ATM inhibitor. In addition, foci formation showed the same dose–response relationship following nucleus irradiation (Figs. 3b and 4b) as that after whole-cell irradiation in the presence of an ATM inhibitor. These findings clearly indicate that ATM activated by energy deposition in the cytoplasm is essential for γ-H2AX foci formation at low radiation doses.

### Analysis of gene expression related to DDRs

Cellular responses, or fates, in the region targeted with a low-dose radiation were clearly different depending on whether the radiation energy was deposited in the cytoplasm. We assessed the relative change in the expression of a gene cluster related to the DNA damage signaling pathway after 30 min of WI-38 nuclei or whole-cell irradiation with 1 Gy microbeams. Cluster analysis revealed drastic differences in gene expression depending on whether the cytoplasm was irradiated (see Supplementary Fig. S1 online). In nucleus-irradiation, we observed that cyclin-dependent kinase inhibitor 1A (*CDKN1A*) expression was upregulated by 2.11 folds, while the expression of ABL proto-oncogene 1, non-receptor tyrosine kinase (*ABL1*), cell division cycle 25C (*CDC25C*), and tumor protein p73 (*TP73*) was downregulated by 6.58, 2.50, and 6.58 folds, respectively, compared with that in whole-cell irradiation (Table 1). These results emphasize the importance of low-dose cytoplasmic irradiation in DDRs.

**Table 1:** List of significantly (> 2 folds) upregulated or downregulated genes in whole-cell-irradiated compared with nucleus-irradiated (1 Gy) WI-38 cells from three independent experiments.

Gene symbol	Fold regulation	p*-*value
*CDKN1A*	2.11	0.00034
*ABL1*	− 6.58	0.0080
*CDC25C*	− 2.50	0.0031
*TP73*	− 6.58	0.0080

Each comparison is performed using pairs of samples from the same batch. p-values are calculated using a Student’s *t* test.

## Discussion

If whole-cell-irradiated and nucleus-irradiated cells represent the same absorbed dose in the nucleus, it is reasonable to assume that the amount and quality of DNA damage are similar. Therefore, our results indicate that energy deposition in the cytoplasm, along with the ensuing response, plays an important role in cell survival. In this study, we developed a method to irradiate most areas of the cellular cytoplasm, excluding the nucleus, with X-rays, and assessed the influence of cytoplasmic irradiation on cell death. As shown in Fig. 2a, we observed an exponential dose-dependent increase in cell mortality (R^2^ = 0.993) following irradiation of the cytoplasm. Conversely, cytoplasmic irradiation also causes remarkable changes to cell proliferation at 0.92 Gy (Fig. 2b). However, the changes in cell proliferation due to cytoplasmic irradiation would have little effect on the determination of cell fate, since cellular viability is greatly enhanced by whole-cell irradiation, wherein both the nucleus and cytoplasm are irradiated, unlike nucleus irradiation, at all doses. Thus, the changes in cell proliferation might be a side effect of the rescue response by energy deposition to the cytoplasm. Although the cytoplasmic radiation targets for cell death induction remain unclear, damage to nuclear DNA is not considered a cause, since γ-H2AX foci were not observed in cells whose cytoplasm were irradiated with high doses of X-rays (Fig. 1d). Furthermore, the linear dose–response relationship for cell death following cytoplasmic irradiation indicates that damage to cytoplasmic targets, or cell death signals from these targets, may increase in a dose-dependent manner. Oxidative stress derived from the accumulation of reactive oxygen species in the cytoplasm and/or radiation-induced damage to the mitochondria may affect cell death. Irradiation of the cytoplasm reportedly induces oxidative nuclear DNA damage, the release of reactive nitrogen species, enhanced lipid peroxidation, increased expression of downstream products generated by free radicals and oxidants, such as cyclooxygenase-2^19,20^, and augmented mitochondrial fission due to high levels of dynamin-related protein 1 (DRP1)^21^. Under oxidative stress conditions, DRP1 localizes to the mitochondrial membrane, where it catalyzes mitochondrial fission and leads to apoptosis. Moreover, DRP1 is also related to necrosis via the mitochondrial permeability transition pore (PTP) opening^22^. These mechanisms are controlled directly by p53 or p53-dependent modulation of microRNAs, which are involved in post-transcriptional regulation^22–25^. Although the dose–response relationship for these mechanisms is not known, the aforementioned evidence suggests that mitochondria may represent one of the cytoplasmic radiation targets responsible for cell death.

The formation of nuclear foci for some type of DSB repair-related proteins is known to increase linearly with increasing radiation dosage and is linked to possible DSB repair mechanisms. Therefore, γ-H2AX has the potential to become a very sensitive marker of both biological dosimetry and the earliest stage of DSB repair^26^. To evaluate the DDR with or without cytoplasmic energy deposition, we assessed the accumulation of γ-H2AX in V79 cells after 30 min of nuclei or whole-cell irradiation. As shown in Fig. 3a–d, foci formation could not be detected following irradiation of the nuclei with X-rays at doses ≤ 2 Gy. In contrast, foci were observed at doses as low as 1 Gy when the whole-cell was irradiated, even though the amount of DSBs induced by the treatment was almost equal in both cases. It is known that the yield of DSBs in mammalian cells is approximately 0.1–0.2 × 10^–10^ Gy^−1^ Da^−1^ in the case of X-rays^27^. Since the DNA mass per human cell is approximately 4 pg, about 20–40 DSBs per 1 Gy would be generated. Targeting the nucleus with a microbeam can deposit radiation energy within it. From the viewpoint of the interaction between the substance, which was the DNA in the present study, and radiation, DSBs were reliably generated under our experimental conditions. As the irradiation dose was controlled only by time, specifically the opening and closing of the high-speed shutter, it was proven that the cell nucleus could reliably be irradiated by our system to observe formation of γ-H2AX foci at high doses. Moreover, 53BP1 accumulated at the target site in the nucleus even at a dose of 1 Gy, as shown in Fig. 5. In addition, significant cell death enhancement in the cells whose nuclei were irradiated with low doses of X-rays, as shown in Fig. 2a, could be supporting evidence for the induction of DNA damage, including DSBs, in this dose range. Therefore, undoubtedly DSBs are induced in DNA when the nucleus is irradiated with 1–2 Gy X-rays. Nevertheless, γ-H2AX foci were not observed when nuclei were irradiated with 1–2 Gy X-ray microbeams in the current study. Notably, γ-H2AX foci cannot be used as a dosimeter when DSB repair mechanism is not fully activated in irradiated cells, since γ-H2AX foci formation is one of the processes of the DSB repair cascade. Our findings suggest that energy deposition in the cytoplasm may enhance the DDR initiated by the appearance of DSBs in the nucleus. Furthermore, this mechanism may be widely conserved in mammalian cells, as indicated by a similar outcome in the normal human fibroblast WI-38 cell line (Fig. 4a–d).

Reportedly, ATM and DNA-dependent protein kinase (DNA-PK) redundantly phosphorylate H2AX following exposure to IR^28–30^. In addition, when cells are in the S/G_2_ phase, ataxia telangiectasia and Rad3-related (ATR) also contributes to H2AX phosphorylation^31^. Therefore, ATM, DNA-PK, and/or ATR may mediate cytoplasmic radiation responses. Following WI-38 nuclei or whole-cell irradiation with 1 Gy microbeams, expression of DNA-PK (*PRKDC* in Supplementary Fig. S1) tended to be upregulated (< 2-fold) in whole-cell-irradiated cells, compared with nucleus-irradiated cells*.* Similarly, this trend was also noted for ATM expression (see Supplementary Fig. S1 online). These results suggest that induction of both DNA-PK and ATM may occur when radiation energy is deposited in both the cell nucleus and cytoplasm at low doses. If endogenous DNA-PK and ATM are sufficient for cellular responses to DNA damage, the cell does not need to generate these. Therefore, the induction of DNA-PK and ATM gene expression may be evoked to replenish them. In contrast, as shown in Supplementary Fig. S1, *ATR* tended to be upregulated by < 2 folds following nucleus irradiation, in which radiation energy was not deposited in the cytoplasm. This may indicate a shift to an ATR-mediated cellular response because cells cannot replenish ATM without energy deposition in the cytoplasm, as mentioned above. As shown in Fig. 6, although γ-H2AX foci formation was observed following whole-cell irradiation in the presence of an ATM inhibitor at 5 and 10 Gy, it was not observed at doses ≤ 2 Gy. DNA-PK is able to phosphorylate H2AX without ATM or ATR, since the inhibition of DNA-PK suppresses H2AX phosphorylation, independent of ATM, after 10 Gy of γ-ray irradiation^32^. Our results that ATM inhibition does not suppress H2AX phosphorylation at high doses, are consistent with this report. In other words, DNA-PK contributes to the phosphorylation of H2AX in the higher dose region. Thus, ATM is a principal mediator of cytoplasmic radiation responses in cells irradiated at low doses. Although ATM is predominantly thought to be in the nucleus, it has been shown to localize extensively both in the nucleus and cytoplasm^6,9,10,33^. Based on increasing evidence that radiation-induced ATM translocates from the cytoplasm to the nucleus, it has been proposed that radiation leads to oxidation of the ATM dimer in the cytoplasm, subsequently triggering its dose-dependent monomerization. Resulting ATM monomers are then thought to diffuse into the nucleus to allow DSB recognition^10^. In addition to validating ATM as a cytoplasmic radiation target, our γ-H2AX detection assay appears to confirm the ATM nucleo-shuttling theory.

Low-dose hyper-radiosensitivity (HRS) is defined as the hypersensitivity of cells to a radiation dose lesser than ~ 0.5 Gy and is accompanied by a subsequent increase in induced radioresistance (IRR)^34,35^. This type of dose–response relationship has been observed in whole-cell-irradiated V79 cell survival analysis^13^, as shown in Fig. 2a. Similar relationship has also been reported in several other cell lines^34–36^. Insufficient induction of the DDR in cells irradiated with low doses is thought to be the cause of HRS. Cell mortality in HRS reflects apoptotic cell death caused by a failure to undergo ATM-dependent early-G_2_ phase cell cycle arrest. In contrast, transition in the survival response to IRR reflects activation of the G_2_-phase checkpoint, allowing for extra repair time and increasing cell survival^34–36^. Thus, ATM is a key player in not only the phosphorylation of H2AX, but also the regulation of cell death mechanisms in cells irradiated with low-dose radiation. As shown in Fig. 2a, the HRS/IRR response and the surviving fraction at the peak of IRR were greater in V79 cells whose nuclei alone were irradiated, compared with that noted in V79 cells undergoing whole-cell irradiation^13^. In addition, significant changes in cell proliferation by cytoplasmic irradiation were observed in the dose-region where IRR was induced in the HRS/IRR type dose response in whole-cell-irradiated cells (Fig. 2b). These results suggest that stimulation of the cytoplasm by low-dose radiation could improve cell viability through the suppression of HRS and/or enhancement of IRR via ATM-dependent modulation upstream of the DDR cascades. In addition, it has been reported that the cells deficient in Ku70, a key molecule working with DNA-PK on non-homologous end joining (NHEJ), show HRS type dose–response^37^. Exposure to low-dose radiations might cause homologous recombination (HR) to work predominantly, rather than NHEJ, and thus ATM might play an important role in determining cell fate.

**Figure 7:**
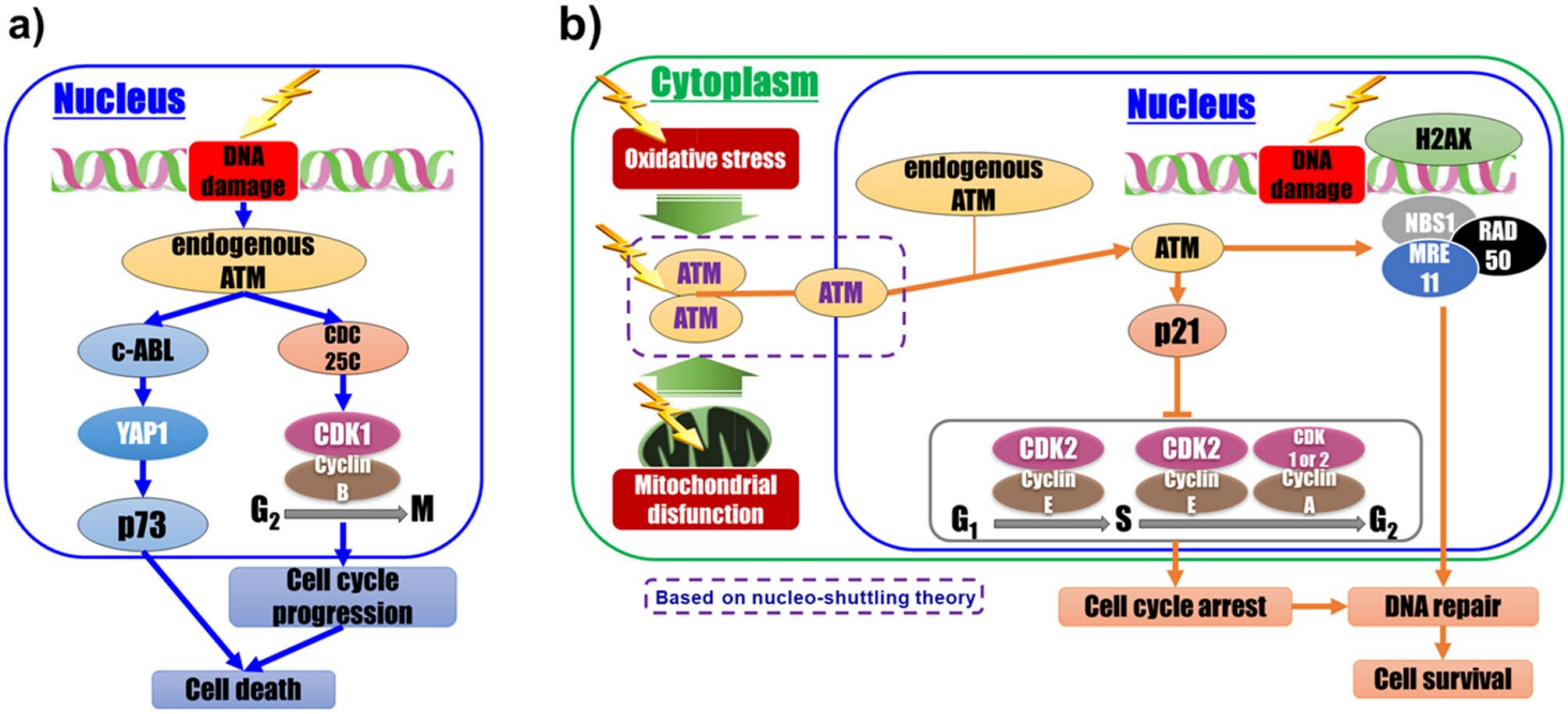
Schematic illustration of the possible cell death-related mechanisms involving cellular signal transduction under exposure to low-dose X-rays. Cell death-related possible mechanisms in nucleus (**a**) and whole-cell (**b**) irradiation with low dose X-ray microbeams. Although functions of the molecules indicated in white color font in the pathway and nucleo-shuttling of ATM were not directly confirmed in this study, they are shown based on the knowledge reported thus far, as described in the discussion.

In this study, we compared the expression of DDR-related genes in WI-38 cells with nucleus and whole-cell irradiation. In the case of whole-cell irradiation, four genes were upregulated or downregulated by > 2 folds, compared with that in cells with nucleus irradiation (Table 1). Primarily, two genes implicated in the induction of apoptosis*, ABL1* and *TP73,* exhibited large differences. TP73 (p73), a homologue of p53, is known as a key regulator of DNA damage-induced cell death^29,38^. In unstressed cells, p73 is degraded by the proteasome^39,40^. Considering the cellular response to genotoxic stress, such as radiation exposure, p73 accumulates and activates several apoptotic target genes^39–42^. *ABL1* encodes c-ABL, a tyrosine kinase retained in the cytoplasm through interaction with the protein 14–3-3ζ^43–45^. Translocation of c-ABL from the cytoplasm to the nucleus is regulated by the phosphorylation of 14-3-3ζ, as mediated by c-Jun N-terminal kinase^45^. In the nucleus, c-ABL is activated directly by ATM to phosphorylate yes-associated protein 1 (YAP1), a co-activator of p73^46–48^. Our results suggest that a majority of cell death mechanisms in the absence of cytoplasmic radiation energy deposition is induced through the apoptotic ATM-c-ABL-YAP1-p73 cascade in cells with the nuclei irradiated with low-dose radiation (Fig. 7a). *CDKN1A* encodes p21, a potent CDK inhibitor, which also functions as a transcriptional target of p53. p53-dependent induction of p21 in irradiated cells results in G_1_-, G_2_-, or S-phase cell cycle arrest^49–53^. Our results suggest that expression of p21 in cells irradiated with low-dose radiation requires cytoplasmic radiation responses (Fig. 7b). Furthermore, CDC25C is known to play an important role in regulating cell cycle transition from G_2_ to M phase^54–59^. The observed upregulation of *CDC25C* in nucleus-irradiated cells may lead to cell cycle progression at the G_2_ checkpoint (Fig. 7a). This cascade may be closely linked to the cell death mechanism in HRS. In addition, the expression patterns of *CDKN1A*, *ABL1*, *CDC25C*, and *TP73* in WI-38 cell populations irradiated with 1 Gy soft X-rays (150 kV, 20 mA) changed in the presence of an ATM-specific inhibitor, exhibiting a trend similar to that noted in WI-38 nucleus-irradiated cells in the microbeam experiments (see Supplementary Fig. S2 online). However, the magnitude of the change in gene expression was smaller than that of the change due to energy deposition in the cytoplasm. Unlike the irradiation of cell populations in the presence of ATM inhibitors, irradiating the nucleus with microbeams results in functional endogenous ATM. Thus, endogenous ATM may activate cell death cascades, even in the absence of an additional ATM supply, through radiation-induced cellular responses that may be induced by energy deposition in the cytoplasm (Fig. 7a,b)*.* Our results strongly suggest that the balance between endogenous and additionally produced ATM plays an important role in cell fate determination via DDR cascades during exposure to low-dose radiation.

In summary, our study clearly demonstrated that intracellular communication between the nucleus and cytoplasm plays an important role in determining cell fate after exposure to IR. This may be determined by a competition between nuclear responses promoting cell death and cytoplasmic responses facilitating DNA repair activity. Although the details of such a mechanism are not sufficiently understood, a likely explanation is the nuclear-cytoplasmic shuttling of ATM, an important upstream moderator/mediator of the DDR. To clarify the mechanism of the competition of nuclear/cytoplasmic responses in the low-dose region, we will analyze the ATM activation status and shuttling in microbeam-irradiated cells in detail as the next step. The significance of this mechanism lies in its potential application in radiosensitization in cancer therapy, whereby radiation doses lower than conventional therapeutic loads are used to efficiently remove neoplastic cells. However, to evaluate the benefits and risks of low-dose radiation to human health, further understanding of the cytoplasmic radiation-sensing mechanisms is required.

## Methods

### Cell culture and sample preparation

Chinese hamster lung V79 (ECACC 86041102) and normal human lung fibroblast WI-38 (ECACC 90020107) cells were obtained from the European Collection of Authenticated Cell Cultures, with low-passage stocks frozen in our laboratory. V79 cells were cultured in α-MEM containing fetal bovine serum (FBS; 10%), penicillin (100 U mL^−1^), streptomycin (100 μg mL^−1^), and 4-(2-hydroxyethyl)-1-piperazineethanesulfonic acid (HEPES; 15 mM). WI-38 cells were cultured in Dulbecco’s Modified Eagle Medium/Ham's F-12 containing FBS (10%), penicillin (100 U mL^−1^), streptomycin (100 μg mL^−1^), and HEPES (15 mM). Cells were incubated in a humidified incubator at 37 °C in an atmosphere containing CO_2_ (5%). For X-ray irradiations, cells were seeded on custom-designed dishes (*Φ* 34 mm, microbeam dish)^13^, of which the bottom was composed of 3 µm-thick polypropylene film (Toray) coated with fibronectin. Before irradiation, cell nuclei were stained with Hoechst 33258 (2 μM) for 1 h, and then the culture medium was replaced with fresh medium (5 mL). For assays using an ATM-specific inhibitor, the culture medium was replaced with fresh medium containing InSolution ATM Kinase inhibitor (20 μM; Merck), and cells were incubated for more than 2 h before irradiation. Next, the same concentration of the inhibitor was added to the exchange medium during and after Hoechst staining.

### X-ray microbeam irradiation

Irradiation with 5.35 keV SR X-ray microbeams was performed with beamline 27B (BL-27B) at the Photon Factory, High Energy Accelerator Research Organization, KEK^12,60^. The tissue penetration depths (1 e^−1^) of the beam and their photoelectrons were 0.29 mm and 0.78 µm, respectively. The transmission of 5.35 keV photons passing through the cells (roughly 10 µm thick) on a base film (3 µm of polypropylene) was > 96%. In our microbeam irradiation system, the position of the beam was fixed, with target irradiation performed on a precise automated stage able to move targets to the position of the beam. The sequences of microbeam irradiation, such as targeting, stage movement, and shutter operation, were automated with dedicated software and the accuracy of movements was < 1 µm^11–13^. During irradiation, we confirmed that all targets were correctly moved to the beam position using captured images; thereby confirming the accuracy of microbeam irradiation of each target. To define the irradiation area in a single V79 cell target, we calculated the average cross-section (nucleus: 168 µm^2^; whole cell: 360 µm^2^), irradiating the nucleus and whole cell with 10 × 10 µm and 50 × 50 µm beams, respectively^13^. In the immunofluorescence assays, populations of V79 cells were uniformly irradiated with 130 × 130 µm beams. For cytoplasmic irradiation, we shielded the nucleus with a micro-shielding X-ray mask containing a gold post (*Φ* 15 µm, t = 20 µm, reduced the intensity of the X-rays by a factor of 1000) on a SiN film (t = 100 µm) (Fig. 1a–c). To confirm the shielding effect of this mask on the nucleus using an immunofluorescence assay, a population of V79 cells was irradiated with a 300 × 300 µm beam with a shielded domain (*Φ* 15 µm) at the center (Fig. 1c). This beam was formed in combination with the X-ray mask and a 500 × 500 µm beam. To irradiate only the cytoplasm of single V79 cells, we combined the 50 × 50 µm beam with the mask (Fig. 1e,f). Given that WI-38 cells are slender compared with V79 cells, beams of 5 × 5 µm, 50 × 50 µm, and 100 × 100 µm or 200 × 200 µm were used for the irradiation of single nuclei, whole cells, and cell populations, respectively. The exposure rate was approximately 7.7 × 10^–3^ C kg^−1^ s^−1^ (9.3 × 10^3^ photons s^−1^ for every 100 µm^2^), which was measured using an AXUV-100 absolute XUV silicon photodiode (International Radiation Detectors). Irradiation of all targets in a sample was generally completed within approximately 20 min to minimize the variability of results, depending on the timing of irradiation, and perform the experiments efficiently. The ‘elapsed time after irradiation’ is designated to start from the time when the irradiated sample is returned to the incubator.

### Calculation of the absorbed dose in the microbeam experiments

When the whole-cell, or population of cells, was uniformly irradiated using X-ray beams, the nuclear and cytoplasmic doses could be considered almost identical. The cellular absorbed dose in the irradiated field (*D*_*cell*_) was calculated using Eq. (1), where *μ*_*en*_/*ρ* is the mass energy absorption coefficient (m^2^ kg^−1^), W is the energy required to generate an ion pair, and *e* is the elementary electric charge^13^.

1 $$ D_{{cell}}  = \frac{{X \times W}}{e} \times \frac{{(\mu _{{en}}{/}\rho )_{{cell}} }}{{(\mu _{{en}}{/}\rho )_{{air}} }} $$

The mean absorbed dose in the nucleus (*D*_*nucleus*_) was calculated using Eq. (2), where *V*_*beam*_ is the volume of the targeted cellular domain in the cell traversed by the beam, and *V*_*nucleus*_ is the total volume of the nucleus^13–15^.

2 $$ D_{{nucleus}}  = \frac{{X \times W}}{e} \times \frac{{(\mu _{{en}}{/}\rho )_{{cell}} }}{{(\mu _{{en}}{/}\rho )_{{air}} }} \times \frac{{V_{{beam}} }}{{V_{{nucleus}} }} $$

The mean absorbed dose in the cytoplasm (*D*_*cytoplasm*_) was calculated using Eq. (3), where *V*_*cytoplasm*_ corresponds to the total volume of the cytoplasm.

3 $$ D_{{cytoplasm}}  = \frac{{X \times W}}{e} \times \frac{{(\mu _{{en}}{/}\rho )_{{cell}} }}{{(\mu _{{en}}{/}\rho )_{{air}} }} \times \frac{{V_{{beam}} }}{{V_{{cytoplasm}} }} $$

In Eq. (3), the value of *V*_*beam*_*/V*_*cytoplasm*_ was ≈ 1, as the cytoplasm was irradiated with the nucleus shielded from the microbeam, whereas *D*_*nucleus*_ could be considered zero due to the insignificant scattering of X-rays and the short range of generated photoelectrons. Conversely, when only the nucleus was targeted, *D*_*cytoplasm*_ could be regarded as almost zero.

### Determination of cell survival

To avoid interference between the colonies originating from single cells, V79 cells were sparsely seeded and incubated for 4 h on the dish and irradiated with microbeams, as previously described^13^. Briefly, the fraction of cells able to survive microbeam irradiation was determined using a single-cell clonogenic assay. Colonies were photographed 60 h after irradiation, and the number of cells in each colony was counted. Colonies containing more than 30 cells were considered survivors^13^. The standard error (*S.E.*) of the surviving fraction was calculated using Eq. (4), where *N* is the total number of cells investigated, *SF* is the surviving fraction of the sample, and *SF*_*0*_ is the surviving fraction of the control^13,14^.

4 $$ S.E. =  \pm \sqrt {\frac{{SF(SF_{0}  + SF - 2SF \times SF_{0} )}}{{N \times SF_{0}^{3} }}} $$

### Immunofluorescence assay

Cells were seeded onto microbeam dishes and incubated overnight to reach ~ 70% confluence at the time of irradiation. Following irradiation, cells were incubated for 30 min in fresh growth medium, washed three times with phosphate-buffered saline (PBS), fixed with Histochoice Tissue Fixative MB (Amresco) for 25 min at 4 °C, and again rinsed with PBS. Cells were treated with PBS supplemented with Triton X-100 (0.1%) at room temperature (20–25 °C) for 20 min, rinsed with PBS supplemented with Tween-20 (PBS-T; 0.01%), and blocked with bovine serum albumin (BSA; 10%) in PBS at room temperature (20–25 °C) for 20 min. After washing with PBS-T once, cells were incubated in BSA (1%) in PBS-T containing an anti-phospho-histone-H2A.X (Ser139) antibody (1:500; Merck) at 4 °C overnight. In case of double-stain for γ-H2AX and 53BP1, cells were fixed as described previously^16^ and co-incubated with anti-p53 binding protein 1 (Ab-1) antibody (1:500; Merck). Cells were then rinsed three times with PBS-T and incubated with PBS containing Alexa-488-conjugated anti-mouse IgG (1:200; Invitrogen) at room temperature (20–25 °C) for 1 h. In the case of double staining, the cells were co-incubated with PBS containing Alexa-568-conjugated anti-rabbit IgG (1:200; Invitrogen). After two washes with PBS-T, cells were stained with 0.01 mg mL^−1^ propidium iodide (PI) solution for 10 min. For double staining, PI staining was omitted. Samples were then washed twice with PBS-T and once with PBS, mounted with SlowFade Gold Antifade Mountant (Invitrogen), and analyzed using an FV300 laser scanning microscope (Olympus) and Image-Pro Plus 7.0 software (Media Cybernetics).

### PCR array analysis

WI-38 cells (5 × 10^3^) were seeded onto a microbeam dish and incubated for 4 h; all cells in the 8 × 8 mm area at the center of the dish (~ 500 cells) were irradiated. Following irradiation, cells were incubated for 30 min in fresh growth medium. Next, the central area of the dish bottom was removed using a sterilized scalpel. Cells from four such sections (~ 2 × 10^3^ cells) were subjected to RNA purification using the miRNeasy Mini Kit (Qiagen) with an automated sample prep system (QIAcube). First-strand cDNA synthesis and pre-amplification of cDNA target templates were performed using the RT^2^ PreAMP cDNA Synthesis Kit and RT^2^ PreAMP Pathway Primer Mix for the human DNA damage signaling pathway (Qiagen) in a Veriti Thermal Cycler (Applied Biosystems). Real-time PCR was performed using RT^2^ Profiler PCR Arrays with a primer set for the human DNA damage signaling pathway (PAHS-029Z; Qiagen) and the StepOnePlus Real-Time PCR system (Applied Biosystems). The expression of gene clusters related to DNA damage signaling in nucleus- and whole-cell-irradiated WI-38 cells was compared using the ΔΔC_T_ method. Each comparison was performed using pairs of samples from the same batch. All experimental procedures were performed according to protocols provided by the manufacturers.

### Statistics and reproducibility

Statistical analyses of data were performed using Microsoft Excel and GraphPad Prism 8.0 software. Data are shown as mean ± standard error of the mean or box-and-whisker plots. The sizes of each data are shown in the figures and tables, or their legends. Statistical significance was analyzed with two-tailed Student’s *t* test, Tukey's multiple comparison test, or Fisher's exact test, and p-values of 0.05 or lesser were considered to denote statistical significance. Experiments were repeated at least three times to ensure reproducibility.

## Supplementary Information

Supplementary Information.

## Data Availability

The data that support the findings of this study are available from the corresponding author upon reasonable request.
