# Supplementary material for: Exposure of the cytoplasm to low-dose X-rays modifies ataxia telangiectasia mutated-mediated DNA damage responses
Source: Sci Rep. 2021 Jul 5;11:13113. doi: 10.1038/s41598-021-92213-z (PMC8255317; doi:10.1038/s41598-021-92213-z)
Supplement: Supplementary file 1 — Supplementary Information. [file 41598_2021_92213_MOESM1_ESM.pdf]

Exposure of the cytoplasm to low-dose X-rays modifies ataxia telangiectasia mutated-mediated DNA damage responses

Munetoshi Maeda[1]*, Masanori Tomita[2], Mika Maeda[1], Hideki Matsumoto[3],
Noriko Usami[4], Kyo Kume[1], and Katsumi Kobayashi[4]

[1] Proton Medical Research Division, Research and Development Department, The Wakasa Wan Energy Research Center, WERC, 64-52-1 Nagatani, Tsuruga-shi, Fukui 914-0192, Japan.

[2] Radiation Safety Research Center, Nuclear Technology Research Laboratory, Central Research Institute of Electric Power Industry, CRIEPI, 2-11-1 Iwado Kita, Komae-shi, Tokyo 201-8511, Japan.

[3] Department of Experimental Radiology and Health Physics, Faculty of Medical Sciences, University of Fukui, 23-3 Matsuoka-Shimoaitsuki, Eiheiji-cho, Fukui 910-1193, Japan.

[4] Photon Factory, Institute of Materials Structure Science, High Energy Accelerator Research Organization, KEK, 1-1 Oho, Tsukuba-shi, Ibaraki 305-0801, Japan.

***Corresponding author:** mmaeda@werc.or.jp

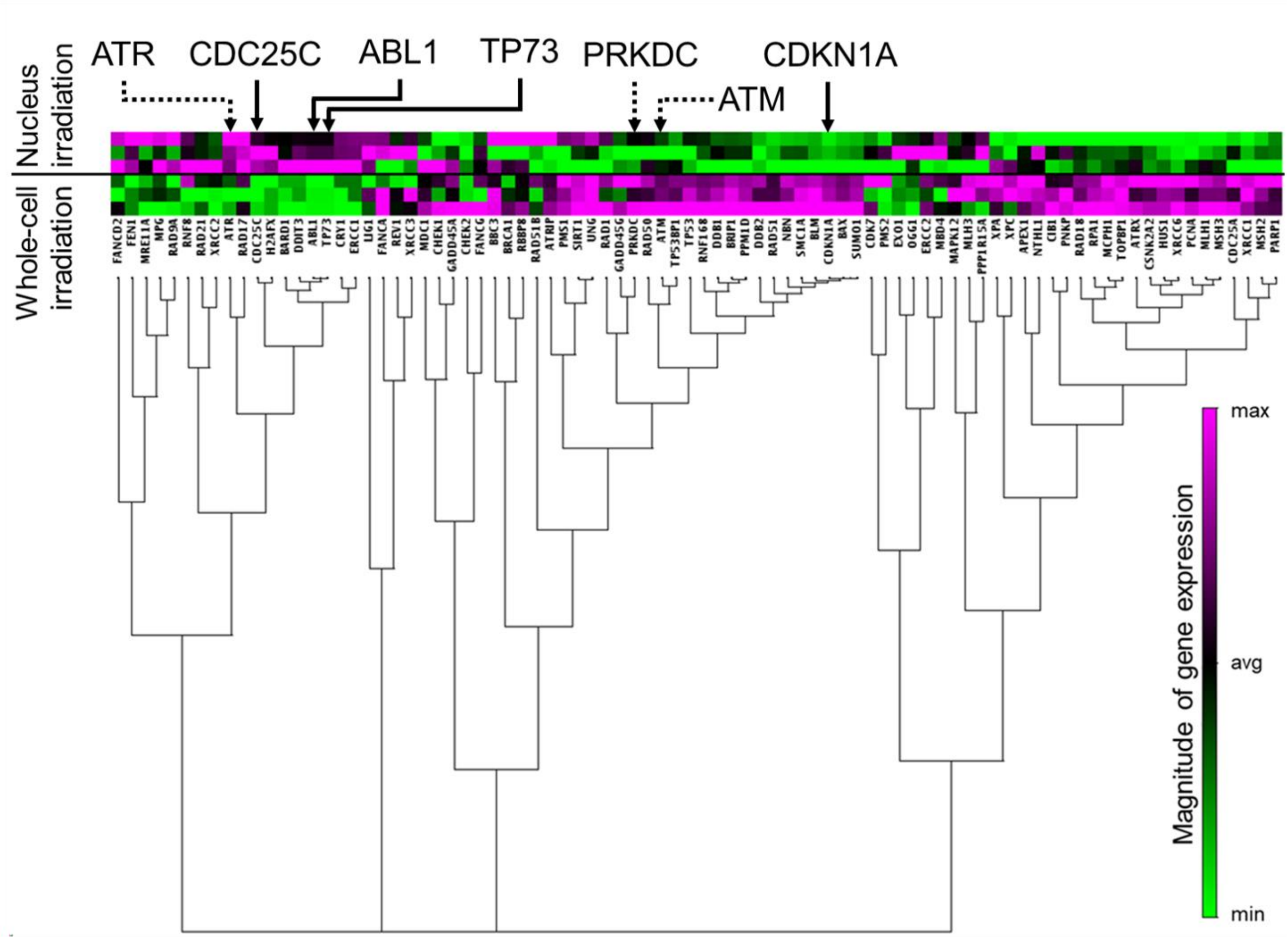

Supplementary Figure S1. Cluster analysis of 84 genes related to DNA damage signaling after 30 min of irradiation.

The nucleus or whole WI-38 cells were irradiated with 1 Gy X-ray microbeams and then analyzed using a PCR array (n = 3). Gene expression was drastically different depending on whether the cytoplasm was irradiated. Although we identified four genes with significant (> 2 folds) differences in expression in whole cells (solid arrows), compared with the nucleus-irradiated cells, these data, including those on the other 80 genes, support our conclusion that the cellular signaling process may be modified via cytoplasmic radiation responses. Dotted arrows indicate the three genes mentioned in the discussion section.

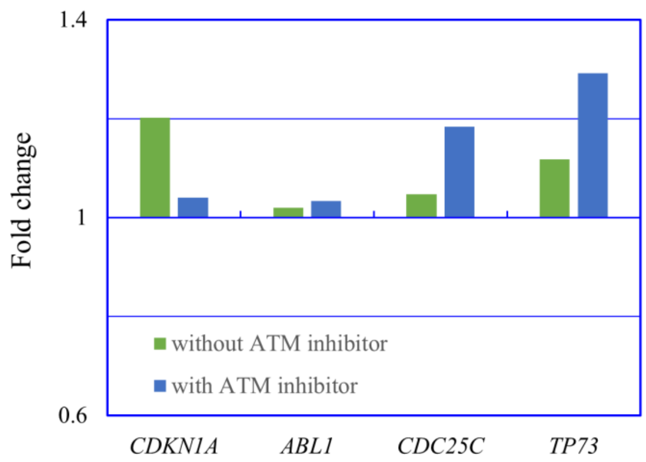

Supplementary Figure S2. Expression of *CDKN1A*, *ABL1*, *CDC25C*, and *TP73* in WI-38 cell populations in the presence or absence of an ataxia telangiectasia mutated (ATM) inhibitor after 30 min of irradiation with a 1 Gy X-ray beam (150 kV, 20 mA) using MBR-1520R-3 (Hitachi Power Solutions, Ibaraki, Japan).

Cells (4.0×10^5) were seeded in T-25 flasks and incubated for 3 days. In the inhibitor-added group, the culture medium was replaced with a medium containing an ATM-specific inhibitor (20 μM, InSolutionTM ATM Kinase inhibitor, Merck), and the cells were incubated for more than 2 h before irradiation. At the time of irradiation, the medium was removed from all samples. Following irradiation, fresh medium (with/without 20 μM ATM inhibitor) was added, and cells were incubated for 30 min in a humidified incubator at 37 °C with an atmosphere containing CO_2 (5%). Next, the cells were harvested and collected, and RNA was purified using the miRNeasy Mini Kit (Qiagen) with an automated sample prep system (QIAcube; Qiagen). Real-time PCR was performed using RT^2 Profiler PCR Arrays with a primer set for the human DNA damage signaling pathway (PAHS-029Z; Qiagen) and the StepOnePlus Real-Time PCR system (Thermo Fisher Scientific). Gene expression under each condition was standardized based on that in the nonirradiated control samples. Due to the addition of an ATM inhibitor, *CDKN1A* expression was downregulated and *ABL1*, *CDC25C*, and *TP73* expression was upregulated. Although this trend is similar to that noted in nucleus-irradiated cells, the magnitude of changes in gene expression was smaller than that of changes due to radiation energy deposition in the cytoplasm.

Supplementary Table S1. Statistical analysis of the viability of the cytoplasm-irradiated V79 cells shown in Fig. 2a.

Comparison pair	Statistical significance	P-value
CTRL vs 0.15 Gy	ns	0.8114
CTRL vs 0.46 Gy	*	0.0351
CTRL vs 0.92 Gy	*	0.0439
CTRL vs 1.85 Gy	***	0.0008
0.15 Gy vs 0.46 Gy	ns	0.0885
0.15 Gy vs 0.92 Gy	***	0.0007
0.15 Gy vs 1.85 Gy	****	<0.0001
0.46 Gy vs 0.92 Gy	ns	0.0796
0.46 Gy vs 1.85 Gy	***	0.0007
0.92 Gy vs 1.85 Gy	ns	0.3146

P-values calculated with Fisher's exact test using the GraphPad Prism 8.0 software (ns: not significant, *: $p < 0.05$, **: $p < 0.01$, ***: $p < 0.001$, ****: $p < 0.0001$).

The R^2 of fitting line calculated with the GraphPad Prism 8.0 software was 0.9930.

Supplementary Table S2. Statistical analysis of the distribution of number of cells per colony of the cytoplasm-irradiated V79 cells shown in Figure 2b.

Comparison pair	Alive		Dead	
	Statistical significance	P-value	Statistical significance	P-value
CTRL vs 0.15 Gy	ns	0.1306	ns	0.6981
CTRL vs 0.46 Gy	ns	0.9915	ns	0.9940
CTRL vs 0.92 Gy	****	<0.0001	ns	0.9900
CTRL vs 1.85 Gy	ns	0.9967	ns	0.2060
0.15 Gy vs 0.46 Gy	ns	0.4087	ns	0.4682
0.15 Gy vs 0.92 Gy	****	<0.0001	ns	0.4164
0.15 Gy vs 1.85 Gy	ns	0.0639	ns	>0.9999
0.46 Gy vs 0.92 Gy	****	<0.0001	ns	>0.9999
0.46 Gy vs 1.85 Gy	ns	0.9420	*	0.0443
0.92 Gy vs 1.85 Gy	***	0.0003	*	0.0182

P-values calculated by Tukey's multiple comparison test with GraphPad Prism 8.0 software (ns: not significant, *: $p < 0.05$, **: $p < 0.01$, ***: $p < 0.001$, ****: $p < 0.0001$).

Supplementary Table S3. Statistical analysis of each mean foci number per V79 cell shown in Figure 3c.

Comparison pair	Nucleus irradiation		Whole-cell irradiation	
	Statistical significance	P-value	Statistical significance	P-value
CTRL vs 1.0 Gy	ns	>0.9999	****	<0.0001
CTRL vs 2.0 Gy	ns	0.3466	****	<0.0001
CTRL vs 4.0 Gy	****	<0.0001	****	<0.0001
CTRL vs 6.0 Gy	***	0.0001	****	<0.0001
CTRL vs 8.0 Gy	***	0.0003	****	<0.0001
CTRL vs 10 Gy	*	0.0168	****	<0.0001
1.0 Gy vs 2.0 Gy	ns	0.3466	*	0.0125
1.0 Gy vs 4.0 Gy	****	<0.0001	****	<0.0001
1.0 Gy vs 6.0 Gy	***	0.0001	****	<0.0001
1.0 Gy vs 8.0 Gy	***	0.0003	****	<0.0001
1.0 Gy vs 10 Gy	*	0.0168	ns	0.6807
2.0 Gy vs 4.0 Gy	****	<0.0001	***	0.0006
2.0 Gy vs 6.0 Gy	***	0.0001	**	0.0015
2.0 Gy vs 8.0 Gy	***	0.0002	**	0.0047
2.0 Gy vs 10 Gy	*	0.0133	*	0.0477
4.0 Gy vs 6.0 Gy	ns	0.4522	ns	0.7855
4.0 Gy vs 8.0 Gy	*	0.0351	ns	0.161
4.0 Gy vs 10 Gy	*	0.018	****	<0.0001
6.0 Gy vs 8.0 Gy	ns	0.1257	ns	0.2877
6.0 Gy vs 10 Gy	ns	0.0522	****	<0.0001
8.0 Gy vs 10 Gy	ns	0.3731	****	<0.0001

Comparison pair	Nucleus irradiation vs Whole-cell irradiation	
	Statistical significance	P-value
CTRL vs CTRL	ns	>0.9999
1.0 Gy vs 1.0 Gy	***	0.0006
2.0 Gy vs 2.0 Gy	**	0.002
4.0 Gy vs 4.0 Gy	***	0.0003
6.0 Gy vs 6.0 Gy	***	0.0003
8.0 Gy vs 8.0 Gy	****	<0.0001
10 Gy vs 10 Gy	ns	0.5963

P-values for each pair were calculated with Student's t-test using the GraphPad Prism 8.0 software. (ns: not significant, *: $p < 0.05$, **: $p < 0.01$, ***: $p < 0.001$, ****: $p < 0.0001$)

Supplementary Table S4. Statistical analysis of each mean fluorescence intensity per focus in the V79 cells shown in Figure 3d.

Comparison pair	Nucleus irradiation		Whole-cell irradiation	
	Statistical significance	P-value	Statistical significance	P-value
CTRL vs 1.0 Gy	ns	0.9758	***	0.0003
CTRL vs 2.0 Gy	ns	0.3466	****	<0.0001
CTRL vs 4.0 Gy	***	0.0005	****	<0.0001
CTRL vs 6.0 Gy	***	0.0001	****	<0.0001
CTRL vs 8.0 Gy	*	0.0122	****	<0.0001
CTRL vs 10 Gy	**	0.0086	**	0.0022
1.0 Gy vs 2.0 Gy	ns	0.3466	ns	0.5173
1.0 Gy vs 4.0 Gy	***	0.0005	***	0.0003
1.0 Gy vs 6.0 Gy	***	0.0001	***	0.0005
1.0 Gy vs 8.0 Gy	*	0.0122	***	0.0003
1.0 Gy vs 10 Gy	**	0.0086	**	0.0066
2.0 Gy vs 4.0 Gy	***	0.0004	***	0.0002
2.0 Gy vs 6.0 Gy	***	0.0001	***	0.0004
2.0 Gy vs 8.0 Gy	*	0.0114	***	0.0002
2.0 Gy vs 10 Gy	**	0.0085	**	0.004
4.0 Gy vs 6.0 Gy	*	0.0117	ns	0.1841
4.0 Gy vs 8.0 Gy	ns	0.1598	ns	0.3222
4.0 Gy vs 10 Gy	*	0.0162	**	0.0027
6.0 Gy vs 8.0 Gy	ns	0.8149	ns	0.6645
6.0 Gy vs 10 Gy	*	0.0348	**	0.0029
8.0 Gy vs 10 Gy	*	0.0343	**	0.0028

Comparison pair	Nucleus irradiation vs Whole-cell irradiation	
	Statistical significance	P-value
CTRL vs CTRL	ns	0.9543
1.0 Gy vs 1.0 Gy	**	0.0072
2.0 Gy vs 2.0 Gy	***	0.0002
4.0 Gy vs 4.0 Gy	ns	0.7915
6.0 Gy vs 6.0 Gy	ns	0.0937
8.0 Gy vs 8.0 Gy	ns	0.1957
10 Gy vs 10 Gy	ns	0.0752

P-values for each pair were calculated with Student's t-test using the GraphPad Prism 8.0 software (ns: not significant, *: $p < 0.05$, **: $p < 0.01$, ***: $p < 0.001$, ****: $p < 0.0001$).

Supplementary Table S5. Statistical analysis of each mean foci number per WI-38 cell shown in Figure 4c.

Comparison pair	Nucleus irradiation		Whole-cell irradiation	
	Statistical significance	P-value	Statistical significance	P-value
CTRL vs 1.0 Gy	ns	>0.9999	****	<0.0001
CTRL vs 2.0 Gy	ns	0.1039	****	<0.0001
CTRL vs 4.0 Gy	***	0.0002	****	<0.0001
CTRL vs 6.0 Gy	**	0.0099	***	0.0009
CTRL vs 8.0 Gy	****	<0.0001	****	<0.0001
CTRL vs 10 Gy	****	<0.0001	****	<0.0001
1.0 Gy vs 2.0 Gy	ns	0.2967	ns	0.1202
1.0 Gy vs 4.0 Gy	***	0.0009	*	0.0151
1.0 Gy vs 6.0 Gy	*	0.0106	ns	0.1195
1.0 Gy vs 8.0 Gy	****	<0.0001	ns	0.4917
1.0 Gy vs 10 Gy	****	<0.0001	ns	0.8124
2.0 Gy vs 4.0 Gy	****	<0.0001	ns	0.1175
2.0 Gy vs 6.0 Gy	**	0.0032	ns	0.2502
2.0 Gy vs 8.0 Gy	****	<0.0001	ns	0.1451
2.0 Gy vs 10 Gy	****	<0.0001	ns	0.1850
4.0 Gy vs 6.0 Gy	ns	0.0774	ns	0.4128
4.0 Gy vs 8.0 Gy	***	0.0003	*	0.0155
4.0 Gy vs 10 Gy	****	<0.0001	*	0.0232
6.0 Gy vs 8.0 Gy	ns	0.1387	ns	0.1207
6.0 Gy vs 10 Gy	*	0.0239	ns	0.1381
8.0 Gy vs 10 Gy	ns	0.0562	ns	0.6953

Comparison pair	Nucleus irradiation vs Whole-cell irradiation	
	Statistical significance	P-value
CTRL vs CTRL	ns	0.4455
1.0 Gy vs 1.0 Gy	**	0.0093
2.0 Gy vs 2.0 Gy	****	<0.0001
4.0 Gy vs 4.0 Gy	***	0.0006
6.0 Gy vs 6.0 Gy	ns	0.3041
8.0 Gy vs 8.0 Gy	**	0.0029
10 Gy vs 10 Gy	**	0.0012

P-values for each pair were calculated with Student's t-test using the GraphPad Prism 8.0 software (ns: not significant, *: $p < 0.05$, **: $p < 0.01$, ***: $p < 0.001$, ****: $p < 0.0001$).

Supplementary Table S6. Statistical analysis of each mean fluorescence intensity per focus in WI-38 cells shown in Figure 4d.

Comparison pair	Nucleus irradiation		Whole-cell irradiation	
	Statistical significance	P-value	Statistical significance	P-value
CTRL vs 1.0 Gy	ns	0.5087	****	<0.0001
CTRL vs 2.0 Gy	ns	0.1829	****	<0.0001
CTRL vs 4.0 Gy	ns	0.1293	****	<0.0001
CTRL vs 6.0 Gy	**	0.0035	****	<0.0001
CTRL vs 8.0 Gy	*	0.0439	**	0.0014
CTRL vs 10 Gy	***	0.0005	*	0.0249
1.0 Gy vs 2.0 Gy	ns	0.2967	ns	0.151
1.0 Gy vs 4.0 Gy	ns	0.1102	**	0.0011
1.0 Gy vs 6.0 Gy	**	0.0029	*	0.027
1.0 Gy vs 8.0 Gy	*	0.0423	ns	0.0999
1.0 Gy vs 10 Gy	***	0.0005	ns	0.1338
2.0 Gy vs 4.0 Gy	ns	0.0689	*	0.0188
2.0 Gy vs 6.0 Gy	**	0.001	ns	0.1354
2.0 Gy vs 8.0 Gy	*	0.0247	ns	0.2151
2.0 Gy vs 10 Gy	***	0.0001	ns	0.1394
4.0 Gy vs 6.0 Gy	ns	0.1601	ns	0.7308
4.0 Gy vs 8.0 Gy	ns	0.0923	ns	0.8097
4.0 Gy vs 10 Gy	***	0.0006	ns	0.0654
6.0 Gy vs 8.0 Gy	ns	0.1957	ns	0.709
6.0 Gy vs 10 Gy	***	0.0008	ns	0.1133
8.0 Gy vs 10 Gy	**	0.003	ns	0.1184

Comparison pair	Nucleus irradiation vs Whole-cell irradiation	
	Statistical significance	P-value
CTRL vs CTRL	ns	0.5202
1.0 Gy vs 1.0 Gy	****	<0.0001
2.0 Gy vs 2.0 Gy	***	0.0002
4.0 Gy vs 4.0 Gy	ns	>0.9999
6.0 Gy vs 6.0 Gy	*	0.037
8.0 Gy vs 8.0 Gy	ns	0.0647
10 Gy vs 10 Gy	ns	0.0819

P-values for each pair were calculated with Student's t-test using the GraphPad Prism 8.0 software (ns: not significant, *: $p < 0.05$, **: $p < 0.01$, ***: $p < 0.001$, ****: $p < 0.0001$).
